# HBcAg-Induced IL-17F drives liver fibrosis in occult HBV infection

**DOI:** 10.1128/spectrum.04235-25

**Published:** 2026-07-17

**Authors:** Bochao Liu, Jieting Huang, Hualong Yang, Qiao Liao, Min Wang, Ru Xu, Zhengang Shan, Huishan Zhong, Fengfang Liao, Tingting Li, Chengyao Li, Huaqin Liang, Yongshui Fu, Xia Rong

**Affiliations:** 1 Guangzhou Blood Center 624661, Guangzhou, China; 2 Institute of Blood Transfusion and Hematology, Guangzhou Medical University 26468, Guangzhou, Guangdong, China; 3 The Key Medical Laboratory of Guangzhou, Guangzhou, Guangdong, China; 4 Department of Transfusion Medicine, School of Laboratory Medicine and Biotechnology, Southern Medical University 70570, Guangzhou, China; 5 Guangzhou First People′s Hospital, Guangzhou Medical University 26468, Guangzhou, Guangdong, China; Engelhardt institute of molecular biology, Moscow, The Russian Federation

**Keywords:** occult HBV infection, IL-17F, liver fibrosis, HBcAg, transcriptome sequencing

## Abstract

While occult hepatitis B virus infection is a known risk factor for progressive liver diseases, the mechanistic basis for this association is poorly understood. To investigate this, we conducted a comparative study involving 40 OBI blood donors and 100 healthy blood donors to assess clinical parameters of liver fibrosis, including serum biomarkers, the FIB-4 index, and liver stiffness measurement. Transcriptome sequencing of peripheral blood mononuclear cells from these donors identified IL-17F as significantly upregulated in the OBI group. Mechanistically, we found that IL-17F was chiefly secreted by CD4^+^ T cells upon HBcAg stimulation and functionally promoted the expression of key fibrosis-related cytokines and markers. Collectively, our study unveils a novel pathway wherein HBcAg induces CD4^+^ T cells to secrete IL-17F, which in turn accelerates liver fibrogenesis, providing a mechanistic basis for OBI-associated liver disease.

**IMPORTANCE:**

This study reveals, for the first time, a novel mechanism by which OBI drives liver fibrosis, demonstrating that HBcAg stimulates CD4^+^ T cells to secrete IL-17F, which in turn activates macrophages and hepatic stellate cells, thereby promoting liver inflammation and fibrosis. This discovery not only elucidates a key molecular pathway underlying OBI-related liver disease progression, but also provides an important theoretical foundation for developing IL-17F-targeted diagnostic approaches and anti-fibrotic therapies.

## INTRODUCTION

After infection with hepatitis B virus (HBV), the virus is typically cleared by the immune system. However, approximately 5% of infected adults may develop a chronic infection, which can progress to chronic hepatitis B (CHB). CHB is defined by a positive hepatitis B virus nucleic acid test persisting for more than 6 months, accompanied by clinical symptoms of chronic hepatitis. This condition may advance to serious liver diseases, such as cirrhosis and hepatocellular carcinoma (HCC). CHB is considered a host-specific immune-mediated liver disorder. Liver injury associated with HBV is likely driven by the accumulation of high levels of viral replication and transcription products within infected hepatocytes (1). During the progression of CHB, hepatitis B surface antigen (HBsAg) has been shown to suppress natural killer (NK) cell activity (2) and interfere with the interferon signaling pathway (3). Additionally, HBsAg can promote hepatocyte apoptosis (4). Thus, HBsAg plays a significant role in the pathogenesis of liver fibrosis, cirrhosis, and HCC in the context of CHB.

Occult hepatitis B infection (OBI) is a distinct form of hepatitis B virus (HBV) infection characterized by the presence of replication-competent HBV DNA, particularly covalently closed circular DNA (cccDNA), in the liver or blood, despite undetectable levels of hepatitis B surface antigen (HBsAg) (5). The mechanisms underlying OBI primarily involve mutations in the HBV S gene, inadequate immune control of HBV by the host, or incomplete response to antiviral therapy in acute or chronic hepatitis B. The prevalence of OBI varies significantly across populations. Among blood donors in China born after 1992, the OBI infection rate is approximately one in 1,494 (6), whereas in high-risk groups, such as individuals coinfected with hepatitis C virus (HCV) or human immunodeficiency virus (HIV), the rate exceeds 10% (7, 8). Due to the absence of HBsAg, OBI is not detectable through routine HBV serological testing. As a result, OBI has long been underrecognized, and infected individuals typically do not receive clinical treatment. However, emerging evidence indicates that OBI carriers not only transmit HBV through blood donations, organ transplants, and other routes (9, 10) but are also associated with progressive liver diseases, including cirrhosis and hepatocellular carcinoma (HCC). Studies reported that OBI was detected in 32% of patients with cryptogenic cirrhosis and 73% of those with cryptogenic HCC (11, 12). Similarly, in the woodchuck model, occult woodchuck hepatitis virus (WHV) infection has been shown to lead to chronic hepatitis and HCC (13, 14).

Liver fibrosis served as the pathological foundation for both liver cirrhosis and hepatocellular carcinoma (HCC). Our preliminary research has revealed that, in addition to cirrhosis and HCC, OBI was also linked to early-stage liver fibrosis. Among patients with liver fibrosis of unknown etiology, those with OBI exhibited significantly higher histological activity index (HAI) and liver stiffness measurement (LSM) values compared to those without OBI. This suggests that OBI may play an important role in the progression of liver fibrosis, although the underlying mechanisms remain to be fully elucidated (15). To gain mechanistic insights into this process, we conducted a comparative study and performed transcriptome sequencing on PBMCs from both OBI blood donors and healthy controls.

## MATERIALS AND METHODS

### Study cohort

A total of 140 age- and gender-matched blood donors were recruited from the Guangzhou Blood Center, with 40 comprising the occult hepatitis B infection (OBI) group (HBsAg^−^/HBV DNA^+^/HBsAb^−^/HBcAb^+^) and 100 serving as healthy controls (HBsAg^−^/HBV DNA^−^/HBsAb^−^/HBcAb^+^). Individuals with other liver diseases, including viral hepatitis, autoimmune, alcoholic, and non-alcoholic fatty liver disease, as well as liver parasites, were excluded. The selection criteria for OBI blood donors were consistent with those described in a previous study (6). This study was approved by the Ethics Committee of the Guangzhou Blood Center, and the research protocol adhered to the ethical principles outlined in the Declaration of Helsinki.

### Biochemical and serological testing

Serum samples of blood donors were collected and tested for liver function indexes (ALT, AST) and liver fibrosis indexes (HA, CG, PIIINP, IV-C, LN), which were quantified with an automatic chemiluminescence analyzer A6200 (Autobio, Zhengzhou, China).

### Liver stiffness measurement

Blood donors were tested for liver stiffness measurement (LSM) using transient elastography (FibroScan Touch 502; Echosens). The LSM values were expressed in kilopascals, and the calculation of LSM value was as described elsewhere (16).

### Sample collection and transcriptome sequencing

Total RNA was extracted from peripheral blood mononuclear cells (PBMCs) of blood donors using the Trizol assay kit. The concentration of the extracted total RNA was measured and used to construct cDNA libraries. The resulting libraries were sequenced on an Illumina NovaSeq 6000 platform. Following sequencing, quality control analysis was performed on the raw data. Principal component analysis (PCA) was then applied to assess the Pearson correlation coefficients between samples and to compare their gene expression profiles. Differential gene expression analysis was carried out using the EdgeR software, with the fold change (FC) used to evaluate the magnitude of expression differences between sample pairs. Based on this analysis, significantly differentially expressed genes (DEGs) were identified by applying thresholds of FDR < 0.05 and |log₂FC| > 1. The RNA-seq data have been deposited in the Gene Expression Omnibus (GEO) database under accession number GSE335534.

### The validation of transcriptome sequencing results by RT-qPCR

Total RNA was extracted from PBMCs using the TRIzol method (Invitrogen) and reverse-transcribed using a Reverse Transcription System according to the manufacturer’s instructions (Roche, Basel, Switzerland). RT-qPCR reactions were performed using SYBR Master Mix following the manufacturer’s protocol (Roche, Basel, Switzerland). The amplification program consisted of an initial denaturation at 95°C for 2 min, followed by 40 cycles of 95°C for 15 s, 55°C for 30 s, and 72°C for 30 s. All quantifications were carried out in triplicate, and glyceraldehyde-3-phosphate dehydrogenase (GAPDH) was used as an internal control. The results are presented as mean  ± standard deviation (SD). The primers specific for IL-17F were forward, 5′-ACGTGAATTCCAGAACCGCT-3′; reverse, 5′-TTGGAGATCGGGCTTCACAC-3′. The primer sequences for other analyzed targets (IL-6, TNF-α, GM-CSF, IL-1α, and GAPDH) were as follows:

IL-6: forward 5′-AGACAGCCACTCACCTCTTCAG-3′, reverse 5′-TTCTGCCAGTGCCTCTTTGCTG-3′;

TNF-α: forward 5′-CCGAGTCTGGGCAGGTCTAC-3′, reverse 5′-TGGGAAGGTTGGATGTTCGT-3′; GM-CSF: forward 5′-TAAGGTCCTGAGGAGGATGTG-3′, reverse 5′-GGGGTGTCCTTATAGAAGCTG-3′;

IL-1α: forward 5′-TGGTAGTAGCAACCAACGGGA-3′, reverse 5′-ACTTTGATTGAGGGCGTCATTC-3′;

GAPDH: forward 5′-GTCTCCTCTGACTTCAACAGCG-3′, reverse 5′-ACCACCCTGTTGCTGTAGCCAA-3′. The fold change (FC) in mRNA levels was calculated as previously described.

### The detection of cytokines by ELISA

The cytokines such as IL-17F, IL-1α, IL-6, TNF-α, CSF, IL-1β, and TGF-β were quantitatively detected using the enzyme-linked immunosorbent assay kit (Dakewe, Beijing, China). According to the instructions of the reagent kit, the OD values of each well were measured at a wavelength of 450 nm. The final OD value for each well was calculated as the OD value at a wavelength of 450 nm minus the mean OD value of the blank wells. The corresponding concentrations of samples were calculated based on the standard curve.

### Isolation and cultivation of macrophages

PBMCs isolated from whole blood of blood donors were cultured in serum-free RPMI-1640 medium for 2 h; suspended cells were removed, and the remaining cells were washed twice with PBS. The remaining adherent cells were cultured in RPMI-1640 complete medium for 7 days to induce spontaneous differentiation of monocytes into macrophages.

### Immunohistochemical staining

LX-2 cells were maintained in high-glucose Dulbecco’s Modified Eagle’s Medium (DMEM, 4.5 g/L glucose) supplemented with 2% fetal bovine serum (FBS), 100 units/mL penicillin, and 100 µg/mL streptomycin. The cells were cultured in a humidified incubator at 37°C with 5% CO₂ and passaged twice weekly using a standard trypsin-EDTA protocol. For experiments, LX-2 cells were seeded in 6-well plates and grown to approximately 70–80% confluence, then treated with 250 ng/mL IL-17F for 48 h, after which the expression of fibrosis markers α-SMA, TIMP1, and COL1A1 was evaluated by immunohistochemistry (IHC) according to a published method (15). In parallel, *in vivo* effects were examined in BALB/c mice (*n* = 5 per group) administered weekly intravenous injections of IL-17F or PBS (vehicle control). One month post-treatment, liver sections from both groups were subsequently analyzed by IHC for α-SMA, Col1α1, and TIMP, following the same established method.

### Immunofluorescence staining

Ten male BALB/c mice (6 weeks old, weighing 20–25 g) were obtained from Guangzhou Medical University. The mice were randomly divided into two groups (*n* = 5 per group). The experimental group received a 2 mL intravenous injection of 10 μg pcDNA3.1-core, while the control group was injected with an equivalent dose of pcDNA3.1-mock. Three days post-injection, liver tissues were harvested from both groups. Immunofluorescence co-staining was then performed on liver sections to detect CD4 (green), IL-17F (red), and DAPI (blue) expression.

### *In vivo* experiments

Mice were divided into three groups: (i) those injected with the pcDNA3.1-core to induce HBcAg expression (HBcAg group), (ii) those injected with pcDNA3.1-mock serving as the negative control, and (iii) those injected with the pcDNA3.1-core followed by administration of an IL-17F antibody at three time points (1 h before and 1 day/3 days after plasmid injection) to inhibit IL-17F function (HBcAg + IL-17F antibody group). At the experimental endpoint, liver tissues were collected from all groups. The mRNA levels of inflammatory cytokines (IL-1α, IL-6, TNF-α, and GM-CSF) were detected by qPCR, while the protein expression of both inflammatory cytokines and fibrosis markers (α-SMA, Col1α1, and TIMP) was evaluated by immunohistochemistry (IHC).

### Statistical analysis

The statistical analysis is performed using SPSS 25.0 software. Continuous variables are expressed as mean ± standard deviation, and independent sample *t*-test is used for comparison between two groups. Categorical variables are expressed as frequencies, and Chi-square test is used for comparison between groups. Differences are considered significant at *P* < 0.05.

## RESULTS

### The comparison of OBI blood donors with healthy blood donors

A total of 40 OBI blood donors and 100 healthy blood donors were enrolled in this study. Their clinical characteristics are summarized in Table 1. No significant differences were observed in sex, age, or BMI between the two groups. However, compared with healthy donors, OBI blood donors showed significantly higher levels of HA, CG, ALT, AST, and FIB-4 index. Liver ultrasound assessment via FibroScan revealed a significant difference in values of liver stiffness measurement (LSM) between the two groups, while no difference was observed in controlled attenuation parameter (CAP) values. These findings suggested an increased risk of liver fibrosis among OBI blood donors.

**TABLE 1:** Clinical characteristics of blood donors with and without OBI

Subjects	OBI blood donors	Healthy blood donors	*P* value
Numbers	40	100	
Sex (male/female)	Male	Male	
Age (year, mean ± SD)	48.5 ± 5.4	51.3 ± 5.9	*P* = 0.113
BMI (kg/m^2^)	22.9 ± 2.2	22.7 ± 2.8	*P* = 0.768
HA (ng/mL)	117.5 ± 63.8	77.7 ± 27.5	*P* < 0.05
PIIINP (ng/mL)	8.3 ± 3.7	7.3 ± 2.2	*P* = 0.318
IV-C (ng/mL)	23.9 ± 8.2	26.5 ± 11.6	*P* = 0.419
LN (ng/mL)	85.9 ± 25.0	79.7 ± 23.1	*P* = 0.415
CG (μg/mL)	2.6 ± 1.3	1.1 ± 0.7	*P* < 0.05
ALT (U/L)	30.2 ± 8.7	20.8 ± 9.7	*P* < 0.01
AST (U/L)	33.9 ± 4.7	24.8 ± 7.8	*P* < 0.001
PLT (10^9^/L)	233.2 ± 47.8	299.6 ± 86.7	*P* < 0.05
FIB-4	1.3 ± 0.3	0.9 ± 0.2	*P* < 0.05
LSM (kPa)	5.6 ± 0.8	3.7 ± 0.7	*P* < 0.001
CAP (dB/m)	206.2 ± 20.8	206.4 ± 20.3	*P* = 0.97

### Transcriptome sequencing results of PBMCs from OBI and healthy blood donors

**Fig 1:**
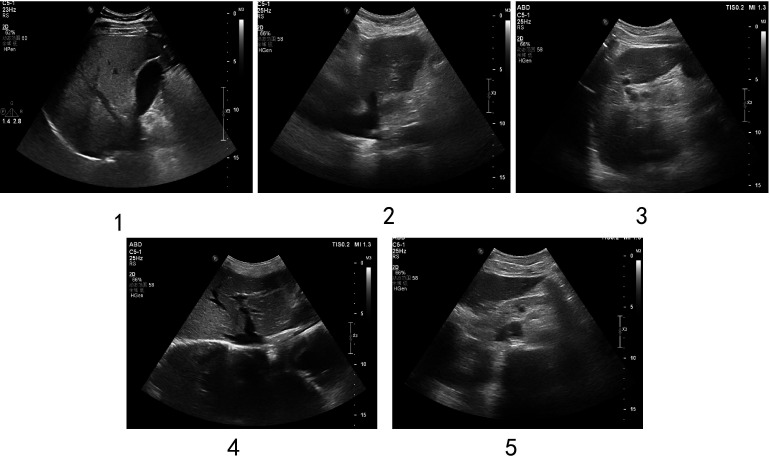
Ultrasound examination revealed an uneven distribution of intrahepatic echoes with thickening and enhanced echogenicity in five abnormal OBI donors.

**Fig 2:**
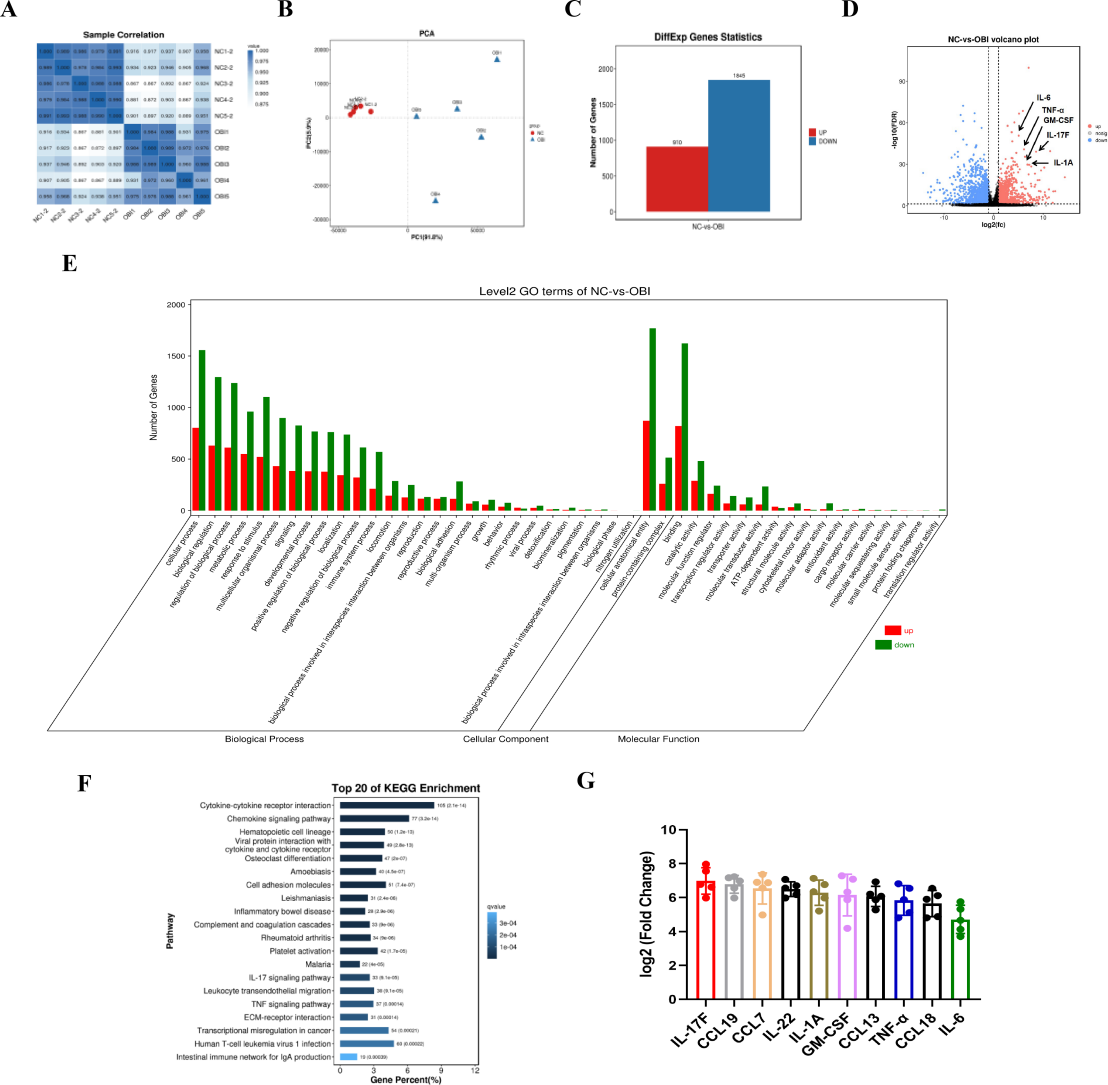
The transcriptome sequencing results of blood donors. (**A**) The Pearson correlation between samples using expression levels in the form of a heatmap. (**B**) The PCA result of respective clustered distributions for two groups. (**C**) DEGs analysis for two groups. (**D**) The fold changes of DEGs between two groups. (**E**) The GO function analysis of DEGs. (**F**) The KEGG pathway analysis of DEGs. (**G**) The top 10 upregulated DEGs related to fibrosis in the OBI group compared with healthy controls.

In light of the increased liver fibrosis susceptibility observed in OBI blood donors, we performed transcriptome sequencing of their PBMCs to identify potential molecular drivers. Samples were obtained from five OBI blood donors with concurrent abnormalities in liver fibrosis markers (≥2 indices), LSM values (≥6.8 kPa), and also moderate-risk FIB-4 index (≥1.3), which were further confirmed by the presence of liver injury through ultrasound detection (Fig. 1), alongside five matched healthy donors as negative controls. These samples were subsequently subjected to transcriptome sequencing. We computed the Pearson correlation coefficients between each pair of samples based on gene expression levels and visualized the correlation matrix as a heatmap (Fig. 2A). The results indicated higher correlations among samples within each group and lower correlations between the two groups. Principal component analysis (PCA) further demonstrated distinct clustering of the two groups (Fig. 2B). Differential gene expression analysis identified 910 upregulated and 1,845 downregulated genes in the OBI group compared to the control group (Fig. 2C). Figure 2D visualized the fold changes of all DEGs, highlighting the magnitude of transcriptional alterations in the OBI group. Functional categorization of the DEGs was performed using Gene Ontology (GO) analysis—covering molecular function, cellular component, and biological process—and Kyoto Encyclopedia of Genes and Genomes (KEGG) pathway analysis (Fig. 2E and F, respectively). Notably, among fibrosis-related DEGs, IL-17F demonstrated the most pronounced upregulation in the OBI group relative to the healthy controls (Fig. 2G).

### The quantitative detection and regression analysis of IL-17F

**Fig 3:**
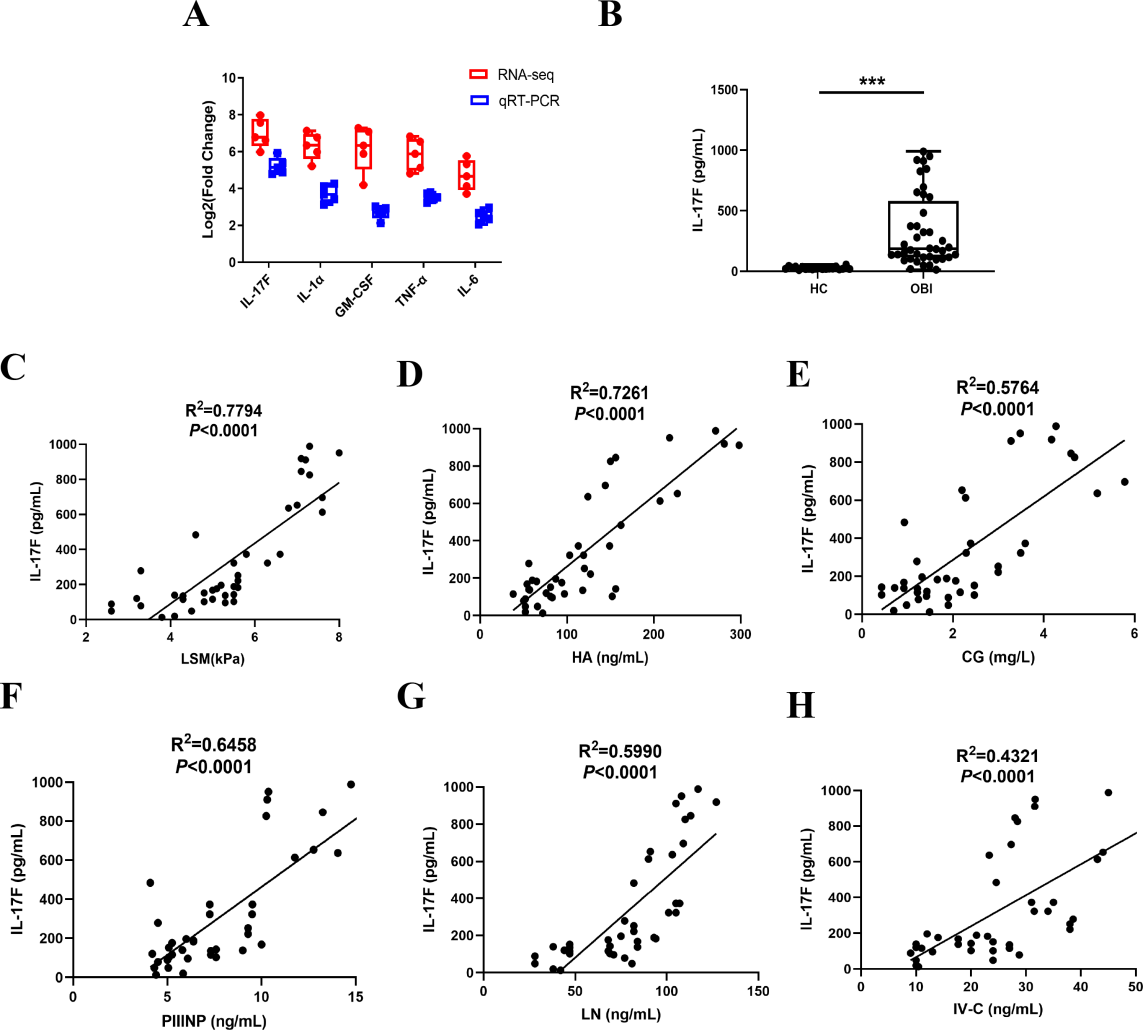
The quantitative detection and regression analysis of IL-17F. (**A**) Relative fold changes of IL-17F mRNA and the expression of IL-17F correlated DEGs in PBMC samples from OBI donors versus healthy controls (HC), as determined by RT-qPCR and RNA-seq. (**B**) Comparison of IL-17F levels in PBMC culture supernatants between the two groups. (**C**) Linear regression analysis between IL-17F levels and LSM values. (**D–H**) Linear regression analysis between IL-17F levels and individual liver fibrosis indexes. In panels **A** and **B**, differences between OBI and HC groups were analyzed using independent two-tailed Student’s *t*-test. For panels **C–H**, Pearson correlation coefficients and linear regression lines are shown. *P < 0.05, **P < 0.01, and ***P < 0.001.

To validate the transcriptome sequencing results, we quantified the relative fold changes of IL-17F mRNA and the expression of IL-17F-correlated DEGs in PBMC samples from OBI donors versus healthy controls (HC) using RT-qPCR and RNA-seq (Fig. 3A). The results demonstrated that IL-17F mRNA levels were significantly higher in OBI donors than in healthy donors, and similar trends were observed for other IL-17F-correlated DEGs. PBMCs obtained from the two groups of blood donors were stimulated for 5 days with a pool of HBcAg peptides (10 μg/mL), which included both HLA class I and class II restricted epitopes (listed in Table S1). The concentration of IL-17F in the culture supernatant was quantitatively measured using ELISA. The results demonstrated that the levels of IL-17F in supernatants from OBI blood donors were significantly higher than those from healthy donors after HBcAg stimulation (Fig. 3B). Furthermore, linear regression analysis was performed to assess the relationship between IL-17F secretion levels from HBcAg-stimulated PBMCs of OBI donors and their corresponding LSM values and liver fibrosis indices. The analysis revealed that IL-17F levels exhibited the strongest correlation with LSM values (*R*^2^ = 0.7794, *P* < 0.0001) and hyaluronic acid (HA) levels (*R*^2^ = 0.7261, *P* < 0.0001) in OBI blood donors (Fig. 3C through H).

### The pro-fibrotic mechanisms of IL-17F

**Fig 4:**
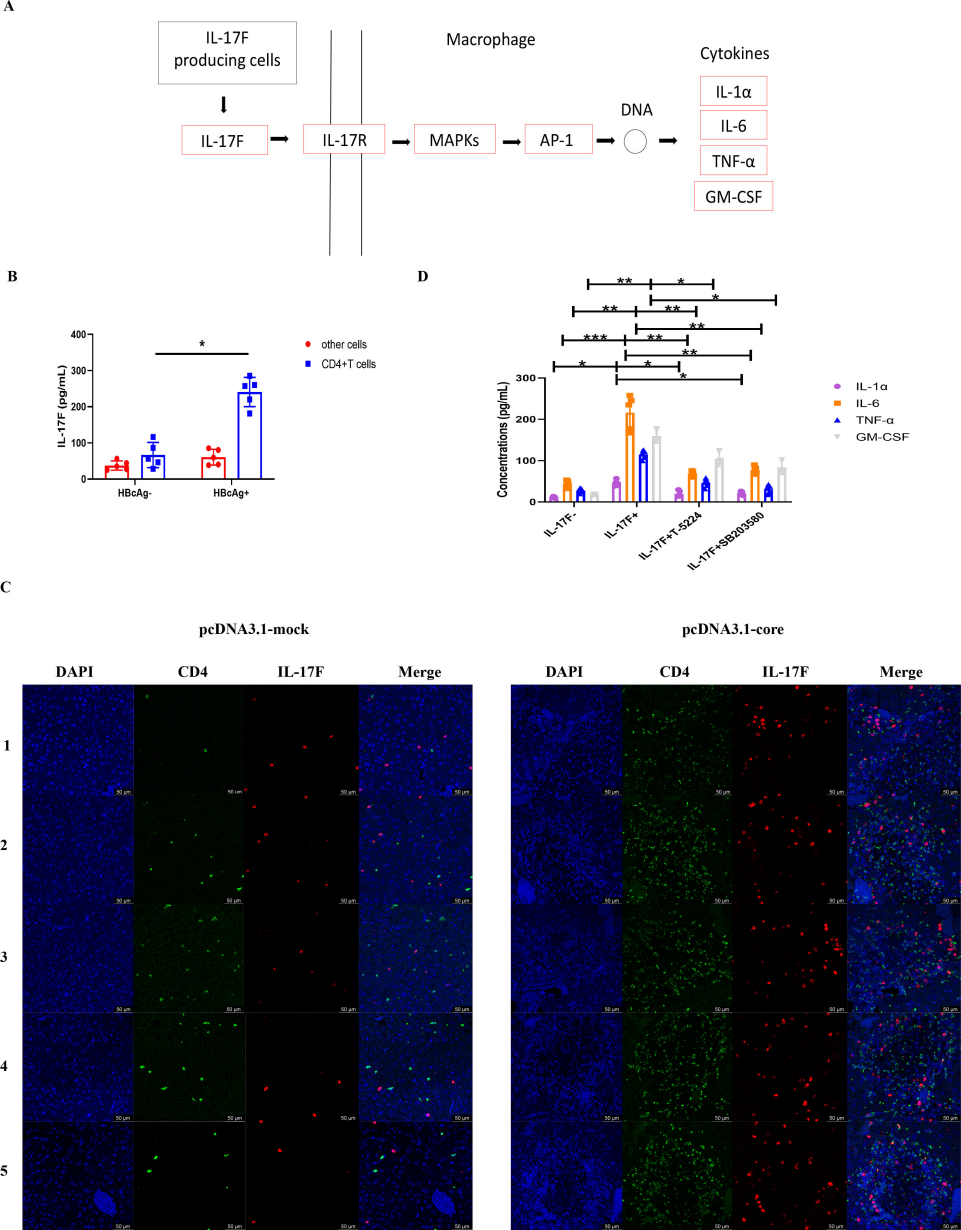
Analysis of the upstream and downstream mechanisms of IL-17F. (**A**) Based on transcriptome sequencing results, the proposed mechanisms of IL-17F action were as follows: IL-17F was secreted and bound to IL-17F receptors on the macrophage surface, leading to activation of downstream MAPK and AP-1 signaling pathways, which in turn modulated transcriptional activity within the nucleus, ultimately resulting in the upregulation of cytokines, such as IL-1α, IL-6, TNF-α, and CSF. Upregulated genes were indicated in red. (**B**) CD4^+^ T cells were isolated from PBMCs of OBI blood donors, and IL-17F secretion levels were quantified by ELISA in both CD4^+^ T cells and the remaining cell fraction, with or without HBcAg stimulation. (**C**) Representative immunofluorescence images showing multiplex staining of CD4^+^ IL-17F^+^ T cells in liver tissue sections (CD4: green; IL-17F: red; nuclei: blue). Liver tissues were obtained from mice injected with either pcDNA3.1-core (*n* = 5) or pcDNA3.1-mock (*n* = 5). (**D**) Upon treatment with 20 μM MAPK inhibitor (SB203580) or 30 μM AP-1 inhibitor (T-5224), macrophages isolated from PBMCs of OBI blood donors were cultured with or without IL-17F stimulation (250 ng/mL) for 72 h, after which the supernatant was collected and analyzed for pro-inflammatory cytokine levels (IL-1α, IL-6, TNF-α, and GM-CSF) by ELISA. *P < 0.05, **P < 0.01, and ***P < 0.001.

By analyzing the differentially expressed genes (DEGs) identified in the transcriptome sequencing results (Fig. 4A), we sought to determine the cellular source of IL-17F. For IL-17F source identification, CD4^+^ T cells were magnetically isolated from OBI donor PBMCs and stimulated with HBcAg peptides (10 μg/mL, 72 h). Subsequent ELISA quantification demonstrated that IL-17F production was predominantly localized to the CD4^+^ T cell population (3.8-fold higher vs non-CD4^+^ cells; *P* < 0.01, Fig. 4B), establishing their central role in OBI-associated IL-17F elevation. This finding was further supported by *in vivo* experiments, where mice injected with pcDNA3.1-core exhibited a higher percentage of CD4^+^IL-17F^+^ T cells in liver tissues than negative controls (Fig. 4C).

Based on transcriptome sequencing results, we predicted that IL-17F might induce macrophages to secrete pro-inflammatory cytokines, such as IL-1α, IL-6, TNF-α, and GM-CSF (Fig. 4A). To verify this, we stimulated OBI-derived macrophages with IL-17F (250 ng/mL, 72 h) and observed a 2.3- to 5.1-fold increase in pro-inflammatory cytokine secretion (IL-1α: *P* < 0.05; IL-6: *P* < 0.001; TNF-α: *P* < 0.01; GM-CSF: *P*<0.01) versus untreated controls by ELISA (Fig. 4D), confirming IL-17F’s role in promoting inflammatory responses. In addition, upon treatment with MAPK inhibitors (SB203580) or AP-1 inhibitors (T-5224), the levels of these pro-inflammatory cytokines secreted by macrophages were markedly reduced, further validating the predictions derived from the transcriptome sequencing data (Fig. 4A).

**Fig 5:**
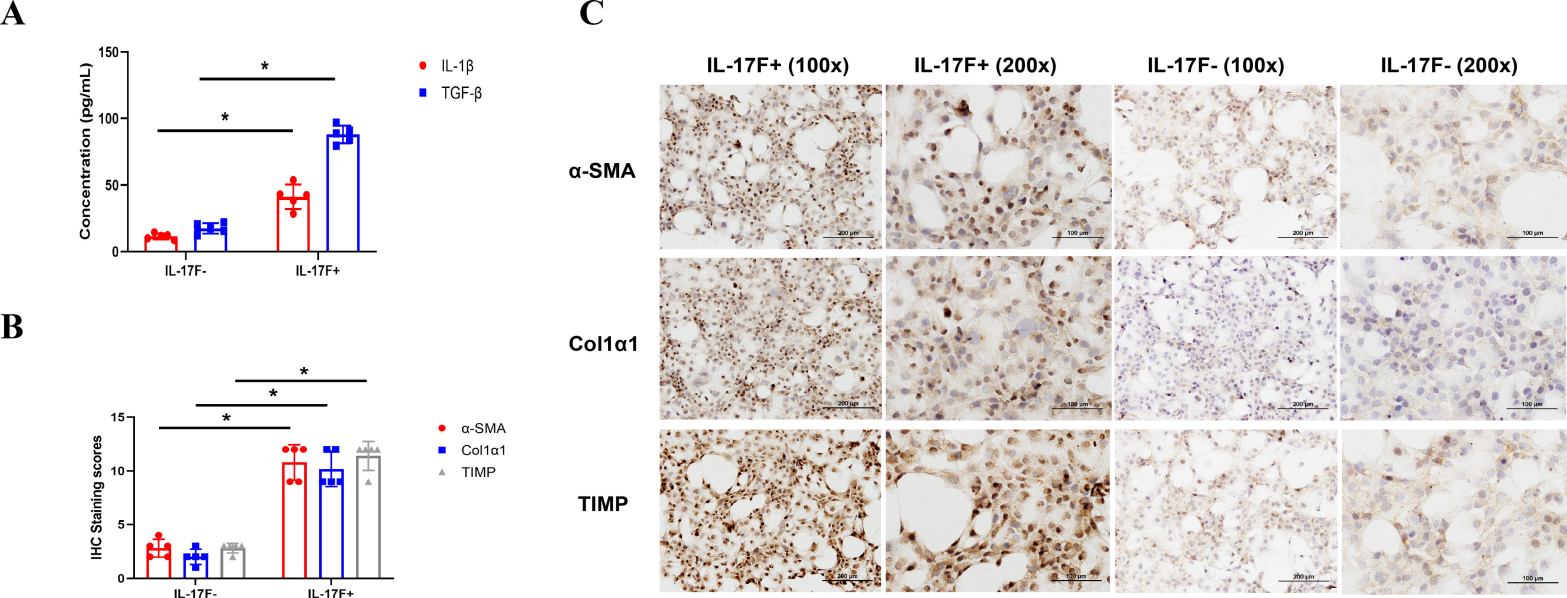
The effect of IL-17F on liver cells. (**A**) Lx-2 cells were stimulated with IL-17F, and the secretion of fibrosis-related cytokines such as IL-1β and TGF-β was tested by ELISA. (**B and C**) The expression of fibrosis indicators, such as α-SMA, Col1α1, and TIMP, was tested by IHC staining. The IHC staining scores were compared between Lx-2 cells with or without IL-17F stimulation (**B**) along with the staining results (**C**). The IHC staining scores were assigned according to the intensity of staining (0, no staining; 1, weak staining; 2, moderate staining; and 3, strong staining) and the extent of stained cells (0, 0%; 1, 1%–10%; 2, 11%–50%; 3, 51%–80%; and 4, 81%–100%). The final score was determined by multiplying the intensity scores by the extent of positive scores of stained cells, with a minimum score of 0 and a maximum score of 12. *P < 0.05, **P < 0.01, and ***P < 0.001.

To further investigate the functional role of IL-17F, we treated Lx-2 cells with IL-17F. Compared to untreated controls, IL-17F stimulation significantly increased the secretion of fibrosis-related cytokines, including IL-1β and TGF-β (Fig. 5A), and upregulated the expression of fibrosis markers, such as α-SMA, Col1α1, and TIMP (Fig. 5B and C).

**Fig 6:**
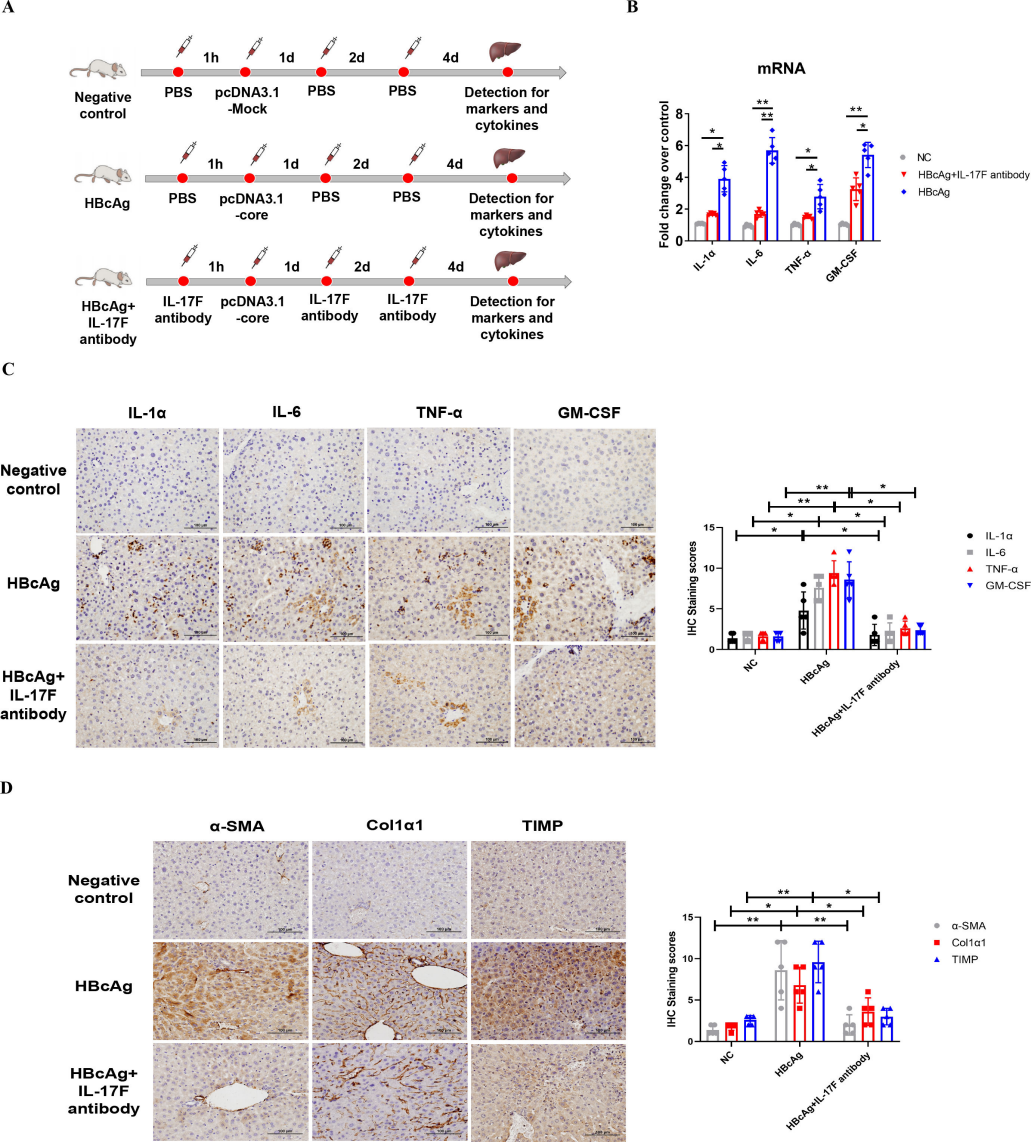
Evaluating the role of IL-17F in an HBcAg mouse model. (**A**) Schematic of the experimental design for the HBcAg mouse model study. Comparison of hepatic mRNA (**B**) and protein expression (**C**) levels of the cytokines IL-1α, IL-6, TNF-α, and GM-CSF among different mouse groups at the experimental endpoint. (**D**) The expression of fibrosis indicators, such as α-SMA, Col1α1, and TIMP, was tested by IHC staining in mouse liver tissues among different groups at the experimental endpoint, and IHC staining scores were compared between three groups. *P < 0.05, **P < 0.01, and ***P < 0.001.

To validate these findings *in vivo*, we employed a hydrodynamic tail vein injection mouse model (Fig. 6A). Mice were divided into three groups: (i) those injected with the pcDNA3.1-core to induce HBcAg expression (HBcAg group), (ii) those injected with pcDNA3.1-mock serving as the negative control, and (iii) those injected with the pcDNA3.1-core followed by administration of an IL-17F antibody at three time points (before and after plasmid injection) to inhibit IL-17F function (HBcAg + IL-17F antibody group). At the experimental endpoint, liver tissues were collected from all groups. Analysis revealed that both the mRNA and protein levels of inflammatory cytokines (IL-1α, IL-6, TNF-α, and GM-CSF) and the expression of fibrosis markers (α-SMA, Col1α1, and TIMP) were significantly upregulated in the liver tissues of the HBcAg group compared with the other two groups (Fig. 6B through D).

## DISCUSSION

Our preliminary study demonstrated that among patients with liver fibrosis of unknown etiology, both the histological activity index (HAI) and liver stiffness measurement (LSM) values were significantly elevated in those with OBI compared to non-OBI individuals, suggesting a potential association between OBI and liver fibrosis progression (15). To further elucidate the mechanisms underlying OBI-related liver fibrosis, we conducted a follow-up study involving 40 OBI blood donors and 100 healthy blood donors. The results showed that both liver fibrosis indices and LSM values were significantly higher in the OBI group than those in the healthy donors, confirming that OBI individuals are indeed at increased risk of liver fibrosis (Table 1).

We performed transcriptome sequencing on PBMCs samples from five OBI blood donors who had at least two abnormal liver fibrosis indicators, abnormal LSM values (≥6.8 kPa), a moderate risk of liver fibrosis (FIB-4 ≥1.3), and also ultrasound evidence of liver damage (Fig. 1), along with five healthy donors as negative controls. The results revealed a significant upregulation of IL-17F in the OBI group (Fig. 2). IL-17F is a member of the IL-17 family, which plays a distinct role in promoting hepatitis progression by recruiting numerous immune cells, thereby amplifying and sustaining fibrotic activation (17). The pathogenic contribution of IL-17 has been established in animal models of liver fibrosis across various etiologies (18, 19). Among IL-17 family members, IL-17A and IL-17F share high amino acid sequence homology and exhibit similar structural and functional characteristics (20). While the role of IL-17A in liver fibrosis has been well documented, that of IL-17F remains less explored. Our transcriptome sequencing findings indicated significant upregulation of IL-17F in OBI donors, suggesting that IL-17F might contribute importantly to liver fibrosis associated with OBI.

In chronic hepatitis B (CHB), HBsAg is a major driver of liver pathology and a sensitive predictor of fibrosis progression (21). In contrast, OBI patients are HBsAg-negative. Recent findings indicated that hepatitis B core antigen (HBcAg) expression was significantly higher in the liver tissues of OBI individuals with cryptogenic liver fibrosis compared to HBsAg levels and correlated positively with fibrosis markers such as hyaluronic acid (HA) (15). These observations suggest that HBcAg may play a critical role in the pathogenesis of OBI-related liver fibrosis.

Accordingly, to confirm the transcriptome sequencing findings, RT-qPCR and RNA-seq were used to determine the relative fold changes of IL-17F mRNA and its correlated DEGs in PBMCs from OBI donors versus healthy controls (HC) (Fig. 3A). OBI donors had significantly higher IL-17F mRNA levels, and other IL-17F-correlated DEGs showed similar trends of upregulation. We further assessed IL-17F levels in supernatants of PBMCs following HBcAg stimulation. The results confirmed that IL-17F expression was indeed higher in OBI donors than in healthy controls (Fig. 3B). Additionally, we performed linear regression analysis between IL-17F levels and liver fibrosis indicators, including liver stiffness measurement (LSM) and hyaluronic acid (HA), in OBI donors. The results showed that IL-17F levels correlated most strongly with HA and LSM values (Fig. 3C through H). This observation was consistent with follow-up data indicating elevated HA and LSM in OBI donors, further supporting an association between IL-17F and liver fibrosis progression.

CD4^+^ T cells isolated from blood donor samples showed significantly elevated IL-17F secretion upon HBcAg stimulation in the OBI group, while no comparable increase was detected in other cell populations (Fig. 4B). This observation was further corroborated by *in vivo* experiments, wherein pcDNA3.1-core injected mice displayed an increased proportion of CD4^+^ IL-17F^+^ T cells in liver tissues relative to controls (Fig. 4C). Together, these results indicate that CD4^+^ T cells are the predominant source of IL-17F in this context.

Through transcriptome sequencing analysis, we identified IL-17F as a potential regulator capable of inducing pro-inflammatory cytokine and chemokine secretion in macrophages, suggesting its involvement in inflammatory responses and subsequent liver fibrosis progression (Fig. 4A). To experimentally validate this observation, we isolated macrophages from human PBMCs and confirmed IL-17F’s pro-fibrotic effects through *in vitro* studies (Fig. 4D). Given that hepatic stellate cells (HSCs) serve as the principal source of extracellular matrix (ECM) deposition during fibrogenesis, we further investigated IL-17F’s impact on LX-2 cells (a human HSC line). IL-17F stimulation significantly upregulated fibrosis-associated cytokines (IL-1β and TGF-β) and enhanced expression of classical fibrotic markers (α-SMA, Col1α1, and TIMP) (Fig. 5A through C). Consistent with these *in vitro* findings, our murine *in vivo* experiments demonstrated that HBcAg upregulated inflammatory and fibrogenic responses, at least in part, through an IL-17F-dependent pathway, as evidenced by the use of an IL-17F-neutralizing antibody that significantly attenuated these responses, collectively establishing the important role of IL-17F in OBI-driven liver fibrosis progression (Fig. 6).

The IL-17 cytokine family, particularly IL-17A and IL-17F, has been increasingly recognized as key regulators of inflammatory and fibrotic processes in various liver diseases (17, 22). In chronic hepatitis B (CHB), IL-17A is known to promote liver fibrosis by activating hepatic stellate cells and recruiting pro-inflammatory cells. Several studies have reported elevated IL-17A expression in CHB patients, which positively correlates with the severity of fibrosis (23–25). Although IL-17A and IL-17F share high amino acid sequence homology and signal through the same IL-17RA/IL-17RC receptor complex (20), their functional roles in liver fibrogenesis may differ depending on the disease context. In contrast to CHB, our transcriptome sequencing data revealed that IL-17F, rather than IL-17A, was the most prominently upregulated IL-17 family member in OBI donors, with expression levels far exceeding those of IL-17A in the same individuals (data not shown). This distinct pattern suggests that IL-17F may serve as a dominant pro-fibrotic driver in OBI, potentially due to HBcAg-specific stimulation of CD4^+^ T cells that preferentially produce IL-17F over IL-17A. These findings underscore the need for further investigation into the unique regulatory mechanisms governing IL-17F expression in occult HBV infection.

While this study provides novel insights into the role of the HBcAg/IL-17F axis in OBI-related liver fibrosis, several limitations should be acknowledged. First, the sample size of OBI donors, particularly those included in the transcriptome sequencing analysis (*n* = 5 per group), remains relatively small. Consequently, the number of OBI donors with significant liver fibrosis (LSM ≥6.8 kPa) in our cohort was extremely limited, precluding a meaningful receiver operating characteristic (ROC) analysis to evaluate the biomarker potential of IL-17F. Although assessing the non-invasive diagnostic value of IL-17F is clinically important, such an analysis would be statistically underpowered and potentially misleading in the current small sample. Future large-scale, prospective cohorts with a sufficient number of OBI subjects presenting with confirmed significant fibrosis are warranted to determine whether IL-17F or its related transcripts could serve as reliable non-invasive biomarkers for OBI-related liver fibrosis. Although our findings were validated through functional assays and animal models, future studies with larger and more diverse cohorts are needed to confirm the generalizability of our results. Second, our mechanistic investigations relied primarily on *in vitro* cell cultures and murine models. While these approaches offer valuable insights, they may not fully recapitulate the complexity of human liver immunology and fibrogenesis. In particular, the use of hydrodynamic injection to induce HBcAg expression in mice does not mirror the natural course of OBI establishment, which involves persistent low-level viral replication and immune adaptation. We acknowledge that this acute plasmid injection model does not fully recapitulate the persistent, low-level viral replication characteristic of true OBI. However, establishing a chronic low-level HBV infection mouse model is technically challenging and time-consuming. Importantly, the primary goal of this study was to validate the causal role of the HBcAg/IL-17F axis in liver fibrogenesis rather than to fully reproduce the entire process of OBI. In future studies, we will employ more physiologically relevant infection models, such as low-titer HBV transfection or transgenic mouse models, to better mimic the human OBI status. Finally, while our data support a pro-fibrotic role for IL-17F, its interaction with other IL-17 family members (e.g., IL-17A) and its contribution to advanced liver disease stages, such as cirrhosis and hepatocellular carcinoma in OBI carriers, remain to be explored.

In conclusion, OBI individuals showed an increased risk of liver fibrosis compared to healthy controls. Follow-up detection and transcriptome sequencing of two groups of blood donors revealed that HBcAg might upregulate IL-17F, which in turn promoted the progression of liver fibrosis during OBI-related fibrogenesis.

## Data Availability

All data are available from the main article and Table S1.

## SUPPLEMENTAL MATERIAL

Table S1 spectrum.04235-25-s0001.docx HLA-I/II restricted HBV core peptides pool.

ASM does not own the copyrights to Supplemental Material that may be linked to, or accessed through, an article. The authors have granted ASM a non-exclusive, world-wide license to publish the Supplemental Material files. Please contact the corresponding author directly for reuse.

## References

[1] Shi Y, Zheng M. 2020. Hepatitis B virus persistence and reactivation. BMJ 370:m2200. doi:10.1136/bmj.m2200 32873599

[2] Du Y, Anastasiou OE, Strunz B, Scheuten J, Bremer B, Kraft A, Kleinsimglinhaus K, Todt D, Broering R, Hardtke-Wolenski M, Wu J, Yang D, Dittmer U, Lu M, Cornberg M, Björkström NK, Khera T, Wedemeyer H. 2021. The impact of hepatitis B surface antigen on natural killer cells in patients with chronic hepatitis B virus infection. Liver Int 41:2046–2058. doi:10.1111/liv.14885 33794040

[3] Lebossé F, Testoni B, Fresquet J, Facchetti F, Galmozzi E, Fournier M, Hervieu V, Berthillon P, Berby F, Bordes I, Durantel D, Levrero M, Lampertico P, Zoulim F. 2017. Intrahepatic innate immune response pathways are downregulated in untreated chronic hepatitis B. J Hepatol 66:897–909. doi:10.1016/j.jhep.2016.12.024 28043874

[4] Li Y, Xia Y, Cheng X, Kleiner DE, Hewitt SM, Sproch J, Li T, Zhuang H, Liang TJ. 2019. Hepatitis B surface antigen activates unfolded protein response in forming ground glass hepatocytes of chronic hepatitis B. Viruses 11:386. doi:10.3390/v11040386 31027244 PMC6520809

[5] Raimondo G, Locarnini S, Pollicino T, Levrero M, Zoulim F, Lok AS. 2019. Update of the statements on biology and clinical impact of occult hepatitis B virus infection. J Hepatol 71:397–408. doi:10.1016/j.jhep.2019.03.034 31004683

[6] Tang X, Allain J-P, Wang H, Rong X, Chen J, Huang K, Xu R, Wang M, Huang J, Liao Q, Shan Z, Luo S, Li T, Li C, Fu Y. 2018. Incidence of hepatitis B virus infection in young Chinese blood donors born after mandatory implementation of neonatal hepatitis B vaccination nationwide. J Viral Hepat 25:1008–1016. doi:10.1111/jvh.12901 29624818

[7] Kao J-H, Chen P-J, Lai M-Y, Chen D-S. 2002. Occult hepatitis B virus infection and clinical outcomes of patients with chronic hepatitis C. J Clin Microbiol 40:4068–4071. doi:10.1128/JCM.40.11.4068-4071.2002 12409376 PMC139665

[8] Re VL, Frank I, Gross R, Dockter J, Linnen JM, Giachetti C, Tebas P, Stern J, Synnestvedt M, Localio AR, Kostman JR, Strom BL. 2007. Prevalence, risk factors, and outcomes for occult hepatitis B virus infection among HIV-infected patients. J Acquir Immune Defic Syndr 44:315–320. doi:10.1097/QAI.0b013e31802ea499 17159655

[9] Candotti D, Assennato SM, Laperche S, Allain J-P, Levicnik-Stezinar S. 2019. Multiple HBV transfusion transmissions from undetected occult infections: revising the minimal infectious dose. Gut 68:313–321. doi:10.1136/gutjnl-2018-316490 29959168

[10] Cholongitas E, Papatheodoridis GV, Burroughs AK. 2010. Liver grafts from anti-hepatitis B core positive donors: a systematic review. J Hepatol 52:272–279. doi:10.1016/j.jhep.2009.11.009 20034693

[11] Chan HL-Y, Tsang SW-C, Leung NW-Y, Tse C-H, Hui Y, Tam JS-L, Chan FK-L, Sung JJ-Y. 2002. Occult HBV infection in cryptogenic liver cirrhosis in an area with high prevalence of HBV infection. Am J Gastroenterology 97:1211–1215. doi:10.1111/j.1572-0241.2002.05706.x 12014730

[12] Wong DKH, Huang FY, Lai CL, Poon RTP, Seto WK, Fung J, Hung IFN, Yuen MF. 2011. Occult hepatitis B infection and HBV replicative activity in patients with cryptogenic cause of hepatocellular carcinoma. Hepatology 54:829–836. doi:10.1002/hep.24551 21809355

[13] Michalak TI, Pardoe IU, Coffin CS, Churchill ND, Freake DS, Smith P, Trelegan CL. 1999. Occult lifelong persistence of infectious hepadnavirus and residual liver inflammation in woodchucks convalescent from acute viral hepatitis. Hepatology 29:928–938. doi:10.1002/hep.510290329 10051500

[14] Mulrooney-Cousins PM, Michalak TI. 2007. Persistent occult hepatitis B virus infection: experimental findings and clinical implications. World J Gastroenterol 13:5682–5686. doi:10.3748/wjg.v13.i43.5682 17963292 PMC4171252

[15] Tang X, Yang L, Zhang P, Wang C, Luo S, Liu B, Fu Y, Candotti D, Allain J-P, Zhang L, Li C, Li T. 2023. Occult hepatitis B virus infection and liver fibrosis in Chinese patients. J Infect Dis 228:1375–1384. doi:10.1093/infdis/jiad140 37170968

[16] Huang L-L, Yu X-P, Li J-L, Lin H-M, Kang N-L, Jiang J-J, Zhu Y-Y, Liu Y-R, Zeng D-W. 2021. Effect of liver inflammation on accuracy of FibroScan device in assessing liver fibrosis stage in patients with chronic hepatitis B virus infection. World J Gastroenterol 27:641–653. doi:10.3748/wjg.v27.i7.641 33642834 PMC7901051

[17] Fabre T, Molina MF, Soucy G, Goulet J-P, Willems B, Villeneuve J-P, Bilodeau M, Shoukry NH. 2018. Type 3 cytokines IL-17A and IL-22 drive TGF-β-dependent liver fibrosis. Sci Immunol 3:28. doi:10.1126/sciimmunol.aar7754 30366940

[18] Hernández-Gea V, Ghiassi-Nejad Z, Rozenfeld R, Gordon R, Fiel MI, Yue Z, Czaja MJ, Friedman SL. 2012. Autophagy releases lipid that promotes fibrogenesis by activated hepatic stellate cells in mice and in human tissues. Gastroenterology 142:938–946. doi:10.1053/j.gastro.2011.12.044 22240484 PMC3439519

[19] Pinzani M. 2015. Pathophysiology of liver fibrosis. Dig Dis 33:492–497. doi:10.1159/000374096 26159264

[20] Wright JF, Bennett F, Li B, Brooks J, Luxenberg DP, Whitters MJ, Tomkinson KN, Fitz LJ, Wolfman NM, Collins M, Dunussi-Joannopoulos K, Chatterjee-Kishore M, Carreno BM. 2008. The human IL-17F/IL-17A heterodimeric cytokine signals through the IL-17RA/IL-17RC receptor complex. J Immunol 181:2799–2805. doi:10.4049/jimmunol.181.4.2799 18684971

[21] Fu TT, Yao Z, Ding H, Xu ZT, Yang MR, Yu JH, Wang WP. 2019. Computer-aided assessment of liver fibrosis progression in patients with chronic hepatitis B: an exploratory research. Zhonghua Yi Xue Za Zhi 99:491–495. doi:10.3760/cma.j.issn.0376-2491.2019.07.003 30786344

[22] Wang L, Chen S, Xu K. 2011. IL-17 expression is correlated with hepatitis B‑related liver diseases and fibrosis. Int J Mol Med 27:385–392. doi:10.3892/ijmm.2011.594 21225222

[23] Paquissi FC. 2017. Immunity and fibrogenesis: the role of Th17/IL-17 Axis in HBV and HCV-induced chronic hepatitis and progression to cirrhosis. Front Immunol 8:1195. doi:10.3389/fimmu.2017.01195 29033929 PMC5626935

[24] Du W-J, Zhen J-H, Zeng Z-Q, Zheng Z-M, Xu Y, Qin L-Y, Chen S-J. 2013. Expression of interleukin-17 associated with disease progression and liver fibrosis with hepatitis B virus infection: IL-17 in HBV infection. Diagn Pathol 8:40. doi:10.1186/1746-1596-8-40 23448394 PMC3598543

[25] Macek Jilkova Z, Afzal S, Marche H, Decaens T, Sturm N, Jouvin-Marche E, Huard B, Marche PN. 2016. Progression of fibrosis in patients with chronic viral hepatitis is associated with IL-17(+) neutrophils. Liver Int 36:1116–1124. doi:10.1111/liv.13060 26749555

