# Supplementary material for: HBcAg-Induced IL-17F drives liver fibrosis in occult HBV infection
Source: Microbiol Spectr. 2026 Jul 17;14(8):e04235-25. doi: 10.1128/spectrum.04235-25 (PMC13435640; doi:10.1128/spectrum.04235-25)
Supplement: Supplementary file 1 [file spectrum.04235-25-s0001.docx]

**Supplementary Table 1. HLA-I/II restricted HBV core peptides pool**

| **HBV Core Peptides** | | | |
| --- | --- | --- | --- |
| HLA class Ⅰ restricted epitopes | | HLA class Ⅱ restricted epitopes | |
| Residues | Amino acid sequence | Residues | Amino acid sequence |
| core11-27 | ASVELLSFLPSDFFPSI | core1-20 | MDIDPYKEFGASVELLSFLP |
| core18-27 | FLPSDFFPSI | core18-27 | FLPSDFFPSI |
| core19-27 | LPSDFFPSI | core28-47 | RDLLDTASALYREALESPEH |
| core88-96 | YVNVNMGLK | core50-69 | PHHTALRQAILCWGELMNLA |
| core107-115 | CLTFGRETV | core111-125 | GRETVLEYLVSFGVW |
| core115-124 | VLEYLVSFGV | core117-131 | EYLVSFGVWIRTPPA |
| core117-125 | EYLVSFGVW | core120-139 | VSFGVWIRTPPAYRPPNAPI |
| core139-148 | ILSTLPETTV | core147-156 | TVVRRRGRSP |
| core141-151 | STLPETTVVRR |  |  |
